# Sedation protocols in non-traumatic SAH (SPRINT-SAH): A cross-sectional survey among German-speaking neurointensivists

**DOI:** 10.3389/fneur.2023.1058804

**Published:** 2023-02-13

**Authors:** Moritz L. Schmidbauer, Hugo Lanz, Andreas Maskos, Timon Putz, Stefan Kunst, Konstantinos Dimitriadis

**Affiliations:** ^1^Department of Neurology, University Hospital LMU Munich, Munich, Germany; ^2^Medizinische Klinik und Poliklinik 1, University Hospital LMU Munich, Munich, Germany; ^3^Institute for Stroke and Dementia Research (ISD), LMU Munich, Munich, Germany

**Keywords:** SAH, deep sedation, intracranial hypertension, monitoring, withdrawal of sedation

## Abstract

**Background:**

In subarachnoid hemorrhage (SAH), titrating sedation to find a balance between wakefulness with the ability to perform valid clinical examinations on the one hand, and deep sedation to minimize secondary brain damage, on the other hand, is challenging. However, data on this topic are scarce, and current guidelines do not provide recommendations for sedation protocols in SAH.

**Methods:**

We designed a web-based, cross-sectional survey for German-speaking neurointensivists to map current standards for the indication and monitoring of sedation, duration of prolonged sedation, and biomarkers for the withdrawal of sedation.

**Results:**

Overall, 17.4% (37/213) of neurointensivists answered the questionnaire. Most of the participants were neurologists (54.1%, 20/37) and exhibited a long-standing experience in intensive care medicine (14.9 years, SD 8.3). Among indications for prolonged sedation in SAH, the control of intracranial pressure (ICP) (94.6%) and status epilepticus (91.9%) were most significant. With regard to further complications in the course of the disease, therapy refractory ICP (45.9%, 17/37) and radiographic surrogates of elevated ICP, such as parenchymal swelling (35.1%, 13/37), were the most relevant topics for experts. Regular awakening trials were performed by 62.2% of neurointensivists (23/37). All participants used clinical examination for the therapeutic monitoring of sedation depth. A total of 83.8% of neurointensivists (31/37) used methods based on electroencephalography. As a mean duration of sedation before attempting an awakening trial in patients with unfavorable biomarkers, neurointensivists suggested 4.5 days (SD 1.8) for good-grade SAH and 5.6 days (SD 2.8) for poor-grade SAH, respectively. Many experts performed cranial imaging before the definite withdrawal of sedation [84.6% (22/26)], and 63.6% (14/22) of the participants required an absence of herniation, space-occupying lesions, or global cerebral edema. The values of ICP tolerated for definite withdrawal were smaller compared to that of awakening trials (17.3 mmHg vs. 22.1 mmHg), and patients were required to stay below the threshold value for several hours (21.3 h, SD 10.7).

**Conclusion:**

Despite the paucity of clear recommendations for sedation management in SAH in the pre-existing literature, we found some level of agreement indicating clinical efficacy for certain clinical practices. By mapping the current standard, this survey may help to identify controversial aspects in the clinical care of SAH and thereby streamline future research.

## 1. Introduction

Spontaneous, non-traumatic subarachnoid hemorrhage (SAH) accounts for 2–7% of strokes and 12–19% of neurointensive care unit (NICU) admissions, with sedation playing a paramount role in its treatment (1–3). However, despite the achievements of the management over the last decades, young age of patients, high mortality, and substantial and lasting impact on functional outcome and quality of life led to a disproportional burden of high disease with the loss of productive life years similar to ischemic stroke (2, 4, 5). Initially, many patients suffer from elevated intracranial pressure (ICP) (6, 7) and require diagnostic angiography as well as surgical or endovascular treatment for potential aneurysms. In the further course of the disease, complications such as cerebral vasospasm, dysfunctions of the cerebral microcirculation, or epileptic seizures may also cause secondary brain damage. Thus, sedation in the acute phase of the disease and prolonged sedation to reduce cerebral metabolism over days are frequently used as therapeutic approaches to manage secondary brain damage and have become an integral part of neurocritical care in the treatment of SAH (8). However, sedation without a robust indication is especially harmful in this cohort as serial clinical neurological examinations are pivotal to detecting delayed cerebral ischemia (DCI), and therapeutic options are extremely time critical (9, 10).

However, data on this topic are scarce, and the current principles in SAH management have predominantly been adopted from traumatic brain injury (TBI) and the general critical care literature (5). Given the unique pathophysiology, general sedation protocols not accounting for the specific challenges in neurocritical care of SAH seem inadequate. So far, the current international guidelines (American Heart Association (AHA), European Stroke Organization (ESO), and Neurocritical Care Society) do not provide specific recommendations for sedation protocols (1, 11, 12). Given this uncertainty, prior studies have documented a great level of heterogeneity in different aspects of SAH management (13, 14). Hernández-Durán et al. (15) have previously also described a heterogeneous practice in Germany, focusing on ventilation parameters, indications for mechanical ventilation and its target values, multimodal neuromonitoring, and the choice of analgesics and sedatives. However, biomarkers relevant to guide sedation in SAH remain unknown.

Overall, it thus remains unclear how the indication and monitoring of sedation, the duration of prolonged sedation, and relevant biomarkers for the withdrawal of sedation are practiced in real life. To further explore the current standards of neurocritical care provided and to identify the research gap for further trials, we designed a cross-sectional survey for German-speaking neurointensivists.

## 2. Materials and methods

### 2.1. Survey design and distribution

With the purpose of characterizing indications, monitoring, duration of prolonged sedation, and relevant biomarkers for the withdrawal of sedation in non-traumatic subarachnoid hemorrhage (SAH), a cross-sectional survey (sedation protocols in non-traumatic SAH, SPRINT-SAH) was initiated. Prolonged sedation was defined as exposure to analgosedation exceeding the conventional limits of procedural sedation. As this may vary among cases, a time threshold was not given. German-speaking neurointensivists were defined as the target population. The list of specialized neurointensive care units provided by the Deutsche Gesellschaft für Neurointensiv- und Notfallmedizin (DGNI) served as the corresponding sample frame (https://www.dgni.de/verzeichnis-neurointensivstationen.html, accessed 19/12/2020). Accordingly, the attending physician responsible for the intensive care unit was the intended reporting unit, and a total of 238 neurointensivists were identified *via* the sample frame. A total of 25 invitations were undeliverable. The survey was answered from 11 January 2021 to 19 February 2021 and was online until 16 February 2022. This study was approved by the institutional ethics committee of Ludwig-Maximilians University Munich, and the requirement for written consent was waived (project number 20-774 KB).

### 2.2. Questionnaire

The predefined aims of the study were operationalized in a questionnaire containing 105 items (Supplementary Table 2). A mixed-method approach was used, combining open- and closed-end questions. Using a Delphi-like process, the initial design of the questionnaire was debated among the members of the Initiative for German NeuroIntensive Trial Engagement, a subdivision of DGNI (IGNITE). Subsequently, new information was integrated and fed back to the panel of experts, resulting in a re-evaluation of the previously made comments. The questionnaire was constructed *via* the web-application Research Electronic Data Capture (REDCAP, Vanderbilt University Tennessee, USA). Patient data were not required, and the respondents remained anonymous. Participants were invited *via* e-mail using unique uniform resource locators (URLs) to prevent multiple reporting. We contacted just one individual per institution to reduce bias by the overrepresentation of single centers. A reminder was issued 2 weeks after the initial invitation. Financial support was not provided. To analyze the intended duration of prolonged sedation relative to various baseline characteristics, the participating neurointensivists were asked to determine the most important biomarker in this context (Tier 1 biomarker). The three most important biomarkers (including Tier 1 biomarker) were given as either favorable or unfavorable to create a fictional clinical scenario. Based on the given baseline information, the respondents were asked to provide a minimum time interval for the duration of sedation.

### 2.3. Data analysis

For statistical analyses, Microsoft Excel (Redmond, USA) and Graph Pad Prism (San Diego, USA) were used. Descriptive statistics are reported in mean and standard deviation (SD). The number of available responses varies among items and is stated in the figure captions as it was not mandatory to answer all items. Boxplots are used to depict certain datasets as a five-number summary. Accordingly, quartiles one and three of the respective data are indicated by the lower and upper boundaries of the box, respectively. A horizontal line within the box represents the median, and the whiskers describe the minimum and maximum of the dataset.

## 3. Results

### 3.1. Demographics of reporting neurointensivists and affiliated institutions

Characteristics of the participating physicians and their corresponding institutions are depicted in Table 1. Overall, 17.4% (37/213) of the identified and contacted neurointensivists answered the questionnaire. The mean age was 50 years (SD 9). More than half of the participants were neurologists [20/37 (54.1%)] and exhibited a long-standing experience in intensive care medicine [14.9 years (SD 8.3)]. Most respondents were practicing at high-volume centers [university hospital 62.2% (23/37) and maximum capacity hospitals 35.1% (13/37)], with an average annual SAH case number of 57 (SD 38.6) at their respective institutions. Most of the intensive care units (ICUs) were led by neurology [12/37 (32.4%)] and used a standard operating procedure (SOP) for the management of SAH [94.6% (35/37)]. However, a smaller proportion had SOPs covering the sedation of patients with SAH in place [67.6% (25/37)].

**Table 1:** Demographics.

**Age (years)**	
*Mean (SD)*	50 (9)
**Institution**	
***N (%)***	
University hospital Maximum capacity hospital Medium capacity hospital Hospital with solely trauma Rehabilitation hospital	23/37 (62.2%) 13/37 (35.1%) 1/37 (2.7%) 0 0
**Annual nontraumatic SAH cases at the institution**	
*Mean (SD)*	57 (38.6)
**Experience in medicine (years)**	
*Mean (SD)*	20.3 (7.3)
**Experience in intensive care medicine (years)**	
*Mean (SD)*	14.9 (8.3)
**Subspecialty**	
*N (%)*	
Neurology Neurosurgery Anesthesiology Internal medicine Others	20/37 (54.1%) 9/37 (24.3%) 8/37 (21.6%) 0 0
**Structure of ICU at the institution**	
*N (%)*	
Neurology led ICU Neurosurgery led ICU Interdisciplinary neurology/neurosurgery led ICU Interdisciplinary anesthesiology/neurology led ICU Interdisciplinary anesthesiology/neurosurgery led ICU Interdisciplinary internal medicine/neurology led ICU Interdisciplinary with neurology and neurosurgery Others	12/37 (32.4%) 1/37 (2.7%) 3/37 (8.1%) 3/37 (8.1%) 8/37 (21.6%) 1/37 (2.7%) 5/37 (13.5%) 4/37 (10.8%)
**Standard operating procedures for**	
*N (%)*	
General management of SAH Sedation of patients with SAH	35/37 (94.6%) 25/37 (67.6%)

SD, standard deviation; N, number; SAH, subarachnoid hemorrhage; ICU, intensive care unit.

### 3.2. Indications and relevant biomarkers for prolonged sedation in SAH

**Figure 1:**
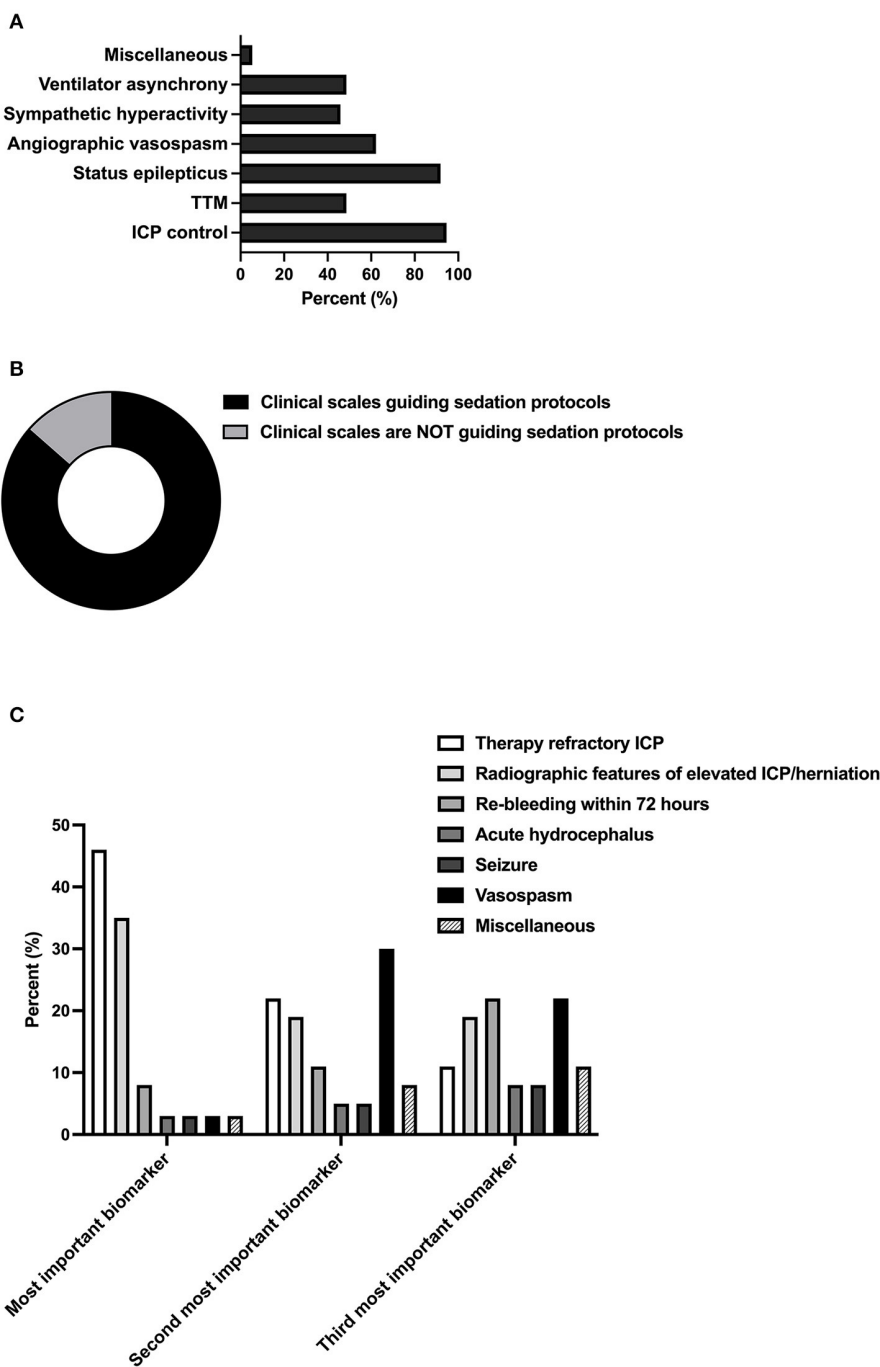
Indications and relevant biomarkers for prolonged sedation. **(A)** Relevance of SAH grading scales (World Federation of Neurological Surgeons (WFNS), Hunt and Hess, modified Fisher scale) to the guidance of sedation protocols [**(B)**; *n* = 37; multiple selection of items]. Indications for prolonged sedation of patients in neurointensive care. Miscellaneous included non-neurointensive care-specific indications (pneumonia, sepsis) [**(C)**; *n* = 37]. Ranking of predefined biomarkers according to their impact on the guidance of sedation protocols. Miscellaneous included ongoing refractory ICP elevation (*n* = 1, most important biomarker), reduction in brain tissue oxygen tension (PtbO2) monitoring (*n* = 2, second most important biomarker) as well as vegetative dysregulation, overall clinical impression, and no relevant other biomarkers (*n* = 2 and *n* = 1, respectively). TTM, targeted temperature management, ICP, intracranial pressure.

Among the general indications for prolonged sedation in SAH, the control of elevated intracranial pressure (ICP) and status epilepticus therapy was deemed to be relevant by 94.6% (35/37) and 91.9% (34/37) of neurointensivists, respectively. However, angiographic vasospasm was evaluated to be an adequate indication for 62.2% of neurointensivists (23/37). Targeted temperature management (TTM) [48.6% (18/37)], ventilator asynchrony [48.6% (18/37)], and sympathetic hyperactivity [45.9% (17/37)] were regarded to be relevant to decision-making by fewer experts (Figure 1A). Surrogates for the severity of disease, as indicated by well-established radiologic and clinical scales (World Federation of Neurological Surgeons (WFNS) grading system and modified Fisher scale), were used by 86.5% (32/37) of all reporting physicians to guide the indication and duration of prolonged sedation (Figure 1B). Respondents were asked to rank a predefined set of biomarkers representing complications in SAH by their relevance for the indication of prolonged sedation. Here, respondents agreed that ICP was the most important biomarker for performing prolonged sedation [therapy refractory ICP measured by invasive monitoring 45.9% (17/37) and radiographic surrogates of elevated ICP such as parenchymal swelling 35.1% (13/37)]. Vasospasm was found to be the second or third most important biomarker by 29.7% (11/37) and 21.6% (8/37), respectively (Figure 1C).

### 3.3. Awakening trials

**Figure 2:**
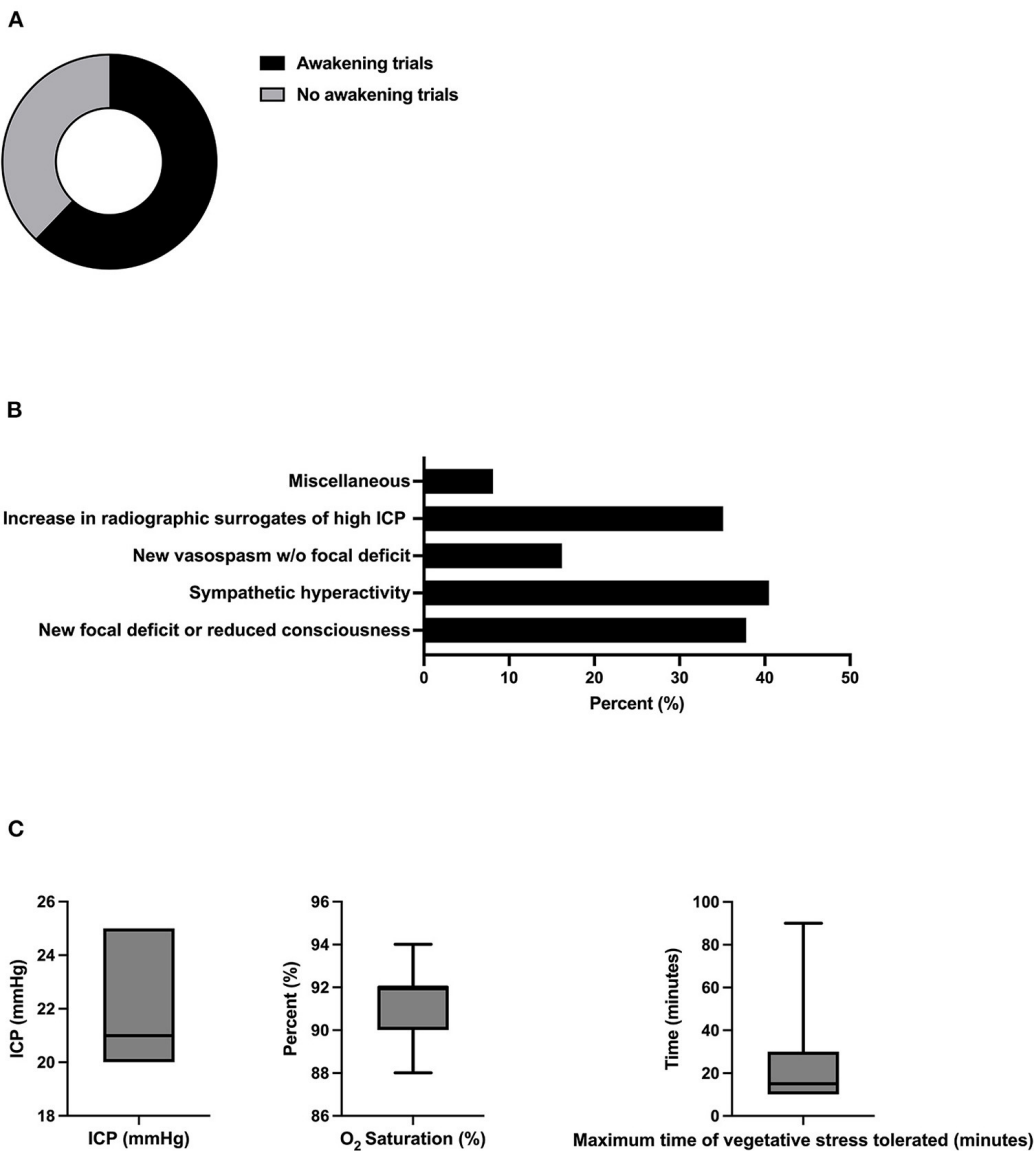
Relevance and safety criteria of daily awakening trials. **(A)** Proportion of experts performing daily awakening trials in patients in SAH (*n* = 37). **(B)** The proportion of experts applying pre-defined safety criteria for interruption of sedation. **(C)** Relevance of predefined safety criteria for interruption of sedation (*n* = 17, multiple selections). Miscellaneous included critical reduction in brain tissue oxygen tension (PtbO2, *n* = 2), status epilepticus (*n* = 1), and hypocapnia (pCO2 < 30mmHg, *n* = 1). **(C)** Thresholds of ICP, vegetative stress, and oxygen saturation. w/o, without, ICP; intracranial pressure.

Regular awakening trials were found to be performed by 62.2% (23/37) of neurointensivists (Figure 2A). Among predefined clinical and radiographic criteria for an interruption of the awakening trial, respondents regarded sympathetic hyperactivity [40.5% (15/37)], new focal neurologic deficits or decreased consciousness [37.8% (14/37)], and an increase in radiologic surrogates of elevated ICP (35.1% (13/37) as the most important variables (Figure 2B). With regard to thresholds of continuously monitored biomarkers, a mean ICP of 22.1 mmHg (SD 2.4) and a mean minimum oxygen saturation of 91.5% (SD 1.8) were tolerated. Vegetative stress reaction upon arousal was tolerated for a mean of 22.5 min (SD 21.3) (Figure 2C).

### 3.4. Monitoring sedation depth

**Figure 3:**
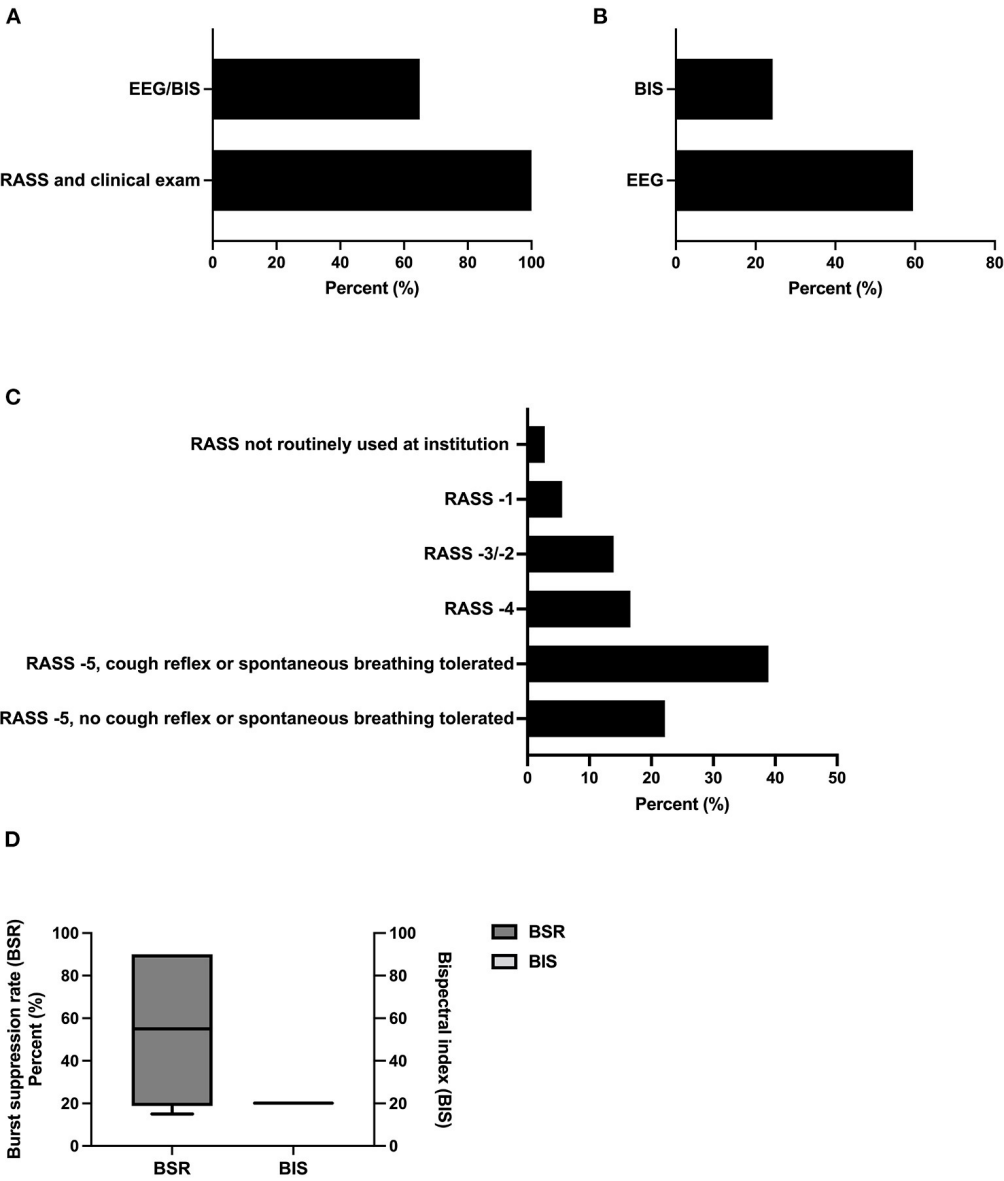
Methods and thresholds for monitoring the level of sedation. **(A)** Methods used for monitoring the level of sedation (*n* = 37, multiple selections). **(B)** Methods used for monitoring *via* EEG-based techniques (*n* = 31, multiple selections). **(C)** Therapeutic targets using RASS as monitoring of the level of sedation (n= 37). **(D)** Therapeutic targets using BIS/EEG as monitoring of the level of sedation (BSR n =6; BIS *n* = 2). BSR was defined as the total time of suppression/epoch length × 100%. BIS, bispectral index, EEG, electroencephalography, BSR, burst suppression ratio.

All participants were found to use clinical examination, including the Richmond Agitation Sedation Scale (RASS), for the therapeutic monitoring of sedation depth. About 83.8% (31/37) used methods based on electroencephalography (EEG) (multichannel EEG 59.5% (22/37) and bispectral index (BIS) 24.3% (9/37) (Figures 3A, B). Target values for RASS were −5 for most participants (59.5% (22/37). In this context, 38.9% (14/37) tolerated cough reflex or spontaneous breathing, whereas 22.2% (8/37) aimed to suppress the latter *via* sedation. For EEG and BIS, a mean burst suppression ratio (BSR) of 54.2% (SD 32.6) and BIS of 20% (SD 0) were reported, respectively (Figure 3C).

### 3.5. Duration of sedation

**Figure 4:**
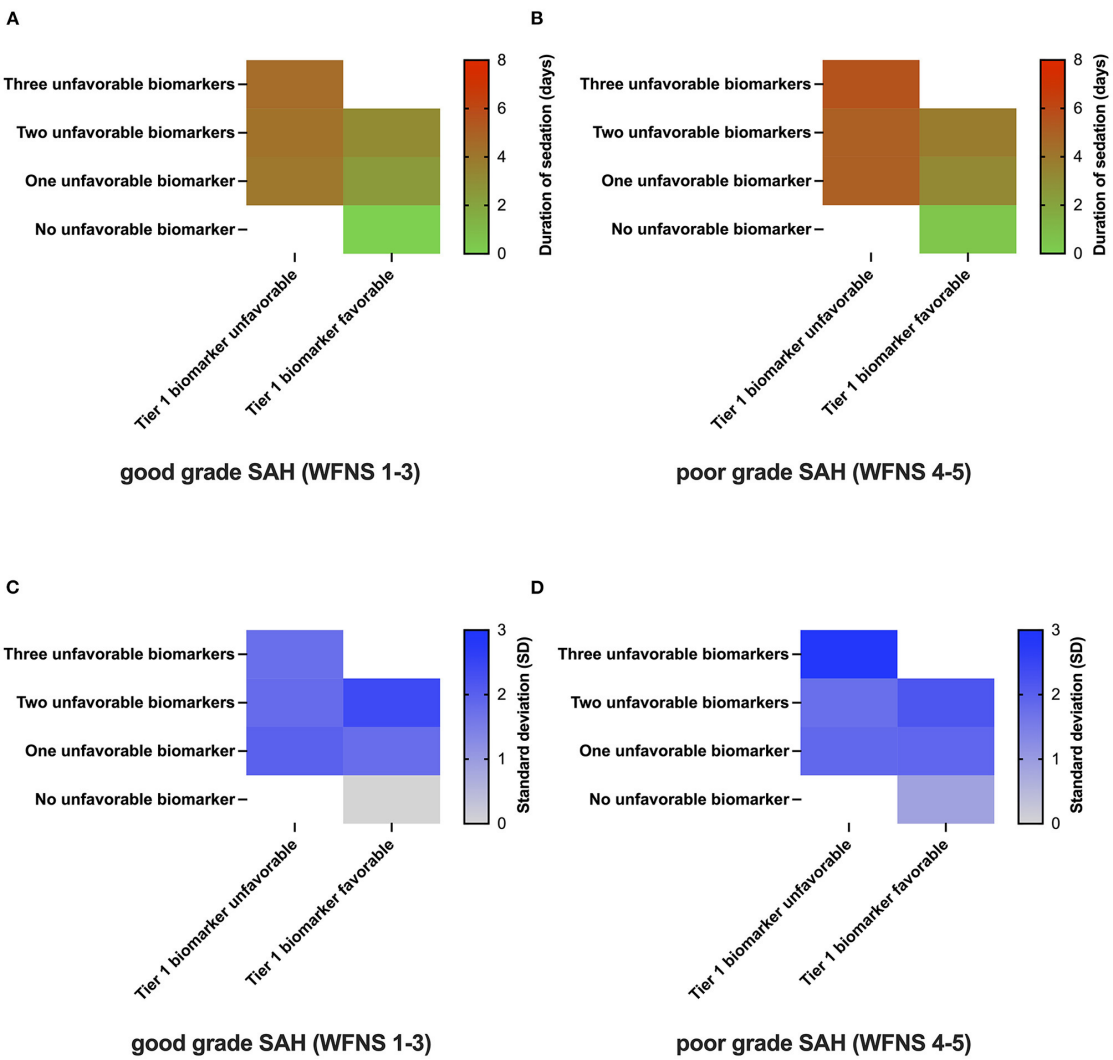
Duration of sedation as a function of SAH biomarkers. The number of unfavorable biomarkers is represented on the y-axis, whereas the most important biomarker's value for the respective answering physician is coded on the x-axis [**(A, B)**; *n* = 7]. Duration of sedation in patients with SAH as a function of the presence/absence of biomarkers in good-grade SAH (WFNS 1-3, **Panel A**) and poor-grade SAH (WFNS 4-5, **Panel B**). The one-gradient heatmap codes for the time of prolonged sedation in days [**(C, D)**; *n* = 7]. The standard deviation of responses for the various clinical scenarios coded by a one-gradient heatmap.

Figure 4 depicts the proposed minimum duration of sedation based on the values of biomarkers individually chosen and ranked by the respondents. Irrespective of the initial disease severity [good-grade SAH WFNS/Hunt and Hess scale (HuH) 1-3: Figure 4A; poor-grade SAH (WFNS/HuH 4–5): Figure 4B], neurointensivists proposed no prolonged sedation over days if all pre-selected biomarkers (possible biomarkers as indicated in Figure 1C, with individual rankings varying from expert to expert) showed a favorable value (WFNS/HuH 1–3: 0 days, SD 0; WFNS/HuH 4–5: 0.4 days, SD 0.9). As the number of unfavorable biomarkers increased, the proposed duration of sedation also increased. Notably, this was more pronounced in the case of unfavorable Tier 1 biomarkers, as well as in poor-grade SAH (Figure 4A vs. Figure 4B). The maximum mean duration of sedation was 4.5 days (SD 1.8) for good-grade SAH and 5.6 days (SD 2.8) for poor-grade SAH, respectively. With respect to drugs used for prolonged sedation, propofol was found to be used more frequently in the early phase of the disease, whereas midazolam/ketamine, volatile anesthetics, and alpha-2 agonists (clonidin, dexmedetomidine) were more commonly used in later phases (Supplementary Table 1).

In order to explore the variance among the different experts involved, the standard deviation of responses was also plotted as a heatmap (Figures 4C, D). Here, a high level of agreement was reached for scenarios with only the favorable values of biomarkers present (WFNS 1-3: SD 0; WFNS 4-5: SD 0.9). The highest standard deviation was reached for poor-grade SAH with all three biomarkers showing unfavorable values (WFNS 1-3: SD 1.8; WFNS 4-5: SD 2.9).

### 3.6. Definite withdrawal of sedation

**Figure 5:**
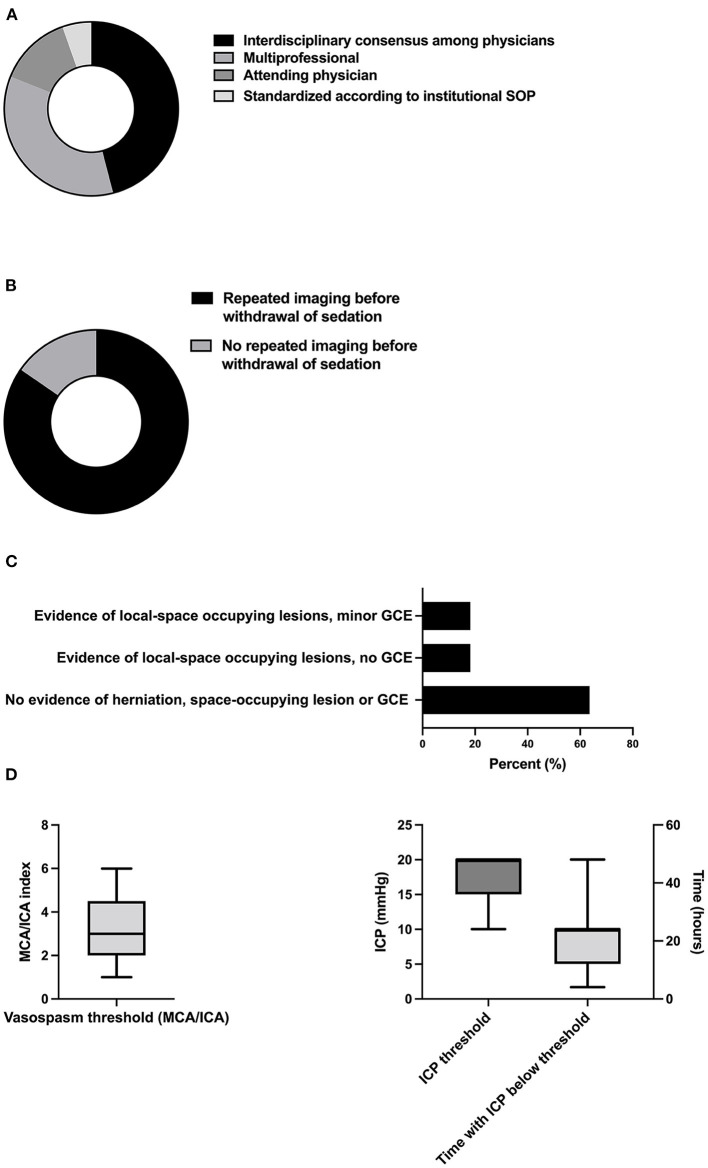
Decision-making and thresholds of definite withdrawal of sedation. **(A)** Responsibilities of decision-making (*n* = 37), **(B)** proportion of neurointensivists demanding CT before the withdrawal of sedation (*n* = 26), **(C)** acceptable radiologic lesion patterns and thresholds for intracranial vasospasm (*n* = 22), and **(D)** and ICP in the context of definite withdrawal of sedation (*n* = 5 with 2 values excluded due to implausible values). SOP, standard operating procedure; GCE, global cerebral edema; MCA, middle cerebral artery; ICA, internal carotid artery; ICP, intracranial pressure.

The vast majority of decisions regarding the definite withdrawal of sedation were not standardized *via* SOPs [94.6% (35/37)] but were rather formed by either physicians in a multidisciplinary fashion [45.9% (17/37)] or by a multiprofessional consensus [35.1% (13/37)], or by the attending physician [13.5% (5/37)] (Figure 5A). Many experts performed cranial imaging before the withdrawal of sedation [84.6% (22/26)] (Figure 5B), and 63.6% (14/22) of the participants required the absence of herniation, space-occupying lesions, or global cerebral edema (GCE). Others accepted space-occupying lesions with [18.2% (4/22)] and without [18.2% (4/22)] evidence of GCE, respectively (Figure 5C). The critical threshold for the middle cerebral artery (MCA)/internal cerebral artery (ICA) index, which describes cerebral vasospasm before the withdrawal of sedation, was determined to be 3.2 (SD 1.8) (Figure 5D). The mean cut off for tolerated ICP before the withdrawal of sedation was 17.3mmHg (SD 3.7). Respondents required the ICP to be below this cutoff for a mean of 21.3 h (SD 10.7) before initiating the definite withdrawal of sedation (Figure 5D).

## 4. Discussion

In this cross-sectional survey of neurointensivists, we evaluated indications and monitoring of prolonged sedation and identified relevant biomarkers and thresholds, ultimately determining the duration of prolonged sedation in non-traumatic SAH. The main findings of this study are as follows: (i) elevated ICP and status epilepticus are the most robust indications for prolonged sedation in neurocritical care, (ii) elevated ICP detected *via* invasive ICP-monitoring and features of elevated ICP on cranial imaging are the most important biomarkers in guiding sedation with regard to duration and depth in patients with SAH, (iii) awakening trials are performed in a substantial part of patients with SAH, (iv) clinical scales and EEG-based monitoring dominate in surveilling sedation depth, (v) the duration of sedation before attempting an awakening trial varies depending on the specific biomarker characteristics with a maximum duration of 5.6 days, and (vi) the definite withdrawal of sedation is often based on expert opinion and requires stable ICP in the preceding period as well as prior cranial imaging.

The results of the survey reflect the literature, which claims critical ICP values to be a widely accepted indication for prolonged sedation in neurointensive care (Figures 1B, C) (16, 17). Elevated ICP, (6, 7, 18) and probably even more or so the overall dose (pressure over time) of ICP, (19) is a frequent and treatable complication in SAH with a profound impact on the patient outcome. However, the rationale for sedation to lower ICP is mainly based on the literature covering traumatic brain injury (20), showing a reduction of ICP with propofol- vs. morphine-based regimens without significant influence on functional outcome (21). Yet, future randomized-controlled trials are deemed to be unethical because of the broad consensus supporting sedation for ICP control. Hence, the current management algorithms are based on expert consensus and list sedation as a Tier 1 strategy (8). Consistent with a prior survey focusing on ventilation and sedatives in SAH care (15), neurointensivists seem to take advantage of propofol's favorable pharmacokinetics and ability to depress the cerebral metabolic rate of oxygen (CMRO2), while midazolam/ketamine, volatile anesthetics, and alpha-2 agonists, such as clonidine and dexmedetomidine, are preferred later on in the clinical course, thereby avoiding complications such as propofol infusion syndrome (PRIS) (Supplementary Table 1). In this regard, the data presented by Hernández-Durán and colleagues suggest a significant correlation between clinical management and the annual SAH case load (15).

Furthermore, the data revealed that status epilepticus is a relevant indication to perform prolonged sedation, which is in line with several trials and international guidelines supporting burst suppression sedation after using adequate doses of benzodiazepines and anticonvulsants over a prolonged period (22, 23).

Apart from ICP, radiographic features of elevated ICP, such as focal lesions, global cerebral edema (GCE) or re-bleeding, were named as SAH-specific indications. As management often includes continuously draining external ventricular drains (EVDs), and thereby limiting the precision of ICP measurements, using radiologic biomarkers as additional information seems reasonable. These expert opinions are backed by data by Zoerle et al. describing early CT lesions as well as re-bleeding to be independent predictors of ICP in patients with SAH (6). Furthermore, GCE has also been shown to be a predictor for ICP-related complications as well as overall morbidity and mortality (24, 25).

Given the remaining controversies regarding awakening trials in neurointensive care, it is of interest that a majority of neurointensivists in our survey stated that they use regular awakening trials in patients with SAH (Figure 2A). On the one hand, it seems plausible that the beneficial effects of minimizing sedation as implemented in general intensive care medicine might also apply to neurointensive care patients. Also, and specific to brain injured patients, only regular interruption of sedation allows valid clinical examinations essential for the detection of new neurological deficits. On the other hand, trials including patients with SAH and traumatic brain injury found that only 40 and 67% were eligible for awakening trials, respectively (26, 27). In addition, approximately one-third of the patients undergoing awakening trials suffered from brain tissue hypoxia or ICP crisis, resulting in the termination of the awakening trial (26). Yet, additional information through clinical assessment of a non-sedated patient could be demonstrated in only one case in a trial involving patients with SAH (26). A study comparing the practice in Scandinavia reported significant heterogeneity, with ~50% of centers never interrupting continuous sedation to enable awakening trials (28). The predefined termination criteria (Figures 2B, C) reported here are similar to the thresholds reported in a landmark trial (26). Furthermore, the recent consensus on neuromonitoring in TBI recommended almost identical ICP thresholds as in our survey (< 22 vs. 22.1 mmHg) (8).

In line with pre-existing data including patients with SAH, all experts covered in this survey used clinical scales, including RASS, to monitor sedation levels (Figure 3A) (15, 29). With regard to the general intensive care population, both RASS and the Sedation-Agitation Scale (SAS) are the most valid and reliable subjective sedation scales (29). Also, there is a correlation between BIS and the above-mentioned sedation scales in patients with acute brain injury (30, 31). Moreover, the use of BIS led to a reduction of sedatives in patients in NICU in a single-center study. However, as some did not exhibit acute brain injury, those with an indication for deep sedation were excluded, and specifics on the respective reason for sedation were not given. Thus, the validity of this study is limited (32). As BIS generates processed EEG data without the physician knowing the variables influencing the algorithm, concerns about the reliability of BIS monitoring have limited its use in patients with brain injury. In line with these considerations, most physicians represented in this survey use EEG without relying on processed data/BIS (Figure 3B). While it is known that CMRO2 is dependent on the dose of the respective sedative used, an optimal range for the burst suppression ratio (BSR) and BIS in prolonged sedation is unknown. As in other domains of neurocritical care, an optimal range of BSR in prolonged sedation fitting to all patients and all indications is implausible. Yet, biomarkers to guide this decision are missing. This level of uncertainty is reflected by the broad interval of responses for BSR (Figure 3C).

For evaluating the duration of sedation, the survey revealed two key aspects (Figure 4). First, biomarkers and hierarchy influencing the duration of sedation vary among experts. Second, the increasing number of biomarkers showing unfavorable values, poor-grade SAH based on WFNS/HuH grading systems, and the presence of unfavorable Tier 1 biomarkers increased the duration of sedation recommended by the participating neurointensivists. Importantly, the duration of sedation reported here represents the minimum duration of sedation without allowing any awakening trial, as the danger of secondary brain injury in the acute phase is deemed too high. Indeed, in longitudinal measurements, intracranial hypertension in the SAH cohort peaks within 2–7 days after ictus, with some patients developing ICP crises as long as 14 days after onset (6). In two high volume centers, the median time patients received sedatives was 6 and 7 days, respectively (19). Thus, our survey contributes to the scarce literature by describing the clinical practice of titrating prolonged sedation in acute SAH based on individual biomarkers. Because data are lacking, there is a high level of uncertainty regarding the most effective protocol for prolonged sedation. This was reflected in our data as an increasing level of disagreement among expert neurointensivists with an increasing number of unfavorable biomarkers was observed.

Similarly, there is no data-driven recommendation regarding criteria for the definite withdrawal of sedation in patients with SAH. Ultimately, the definite interruption of sedation shares features with the awakening trial, which also requires (pre-)defined thresholds prompting the restarting of sedation to be met. However, in contrast to an awakening trial, thresholds for definite interruption tend to be stricter as the interruption of sedation is not temporary. Given these circumstances, many neurointensivists favored prior CT imaging, and most required the absence of herniation, space-occupying lesions, or GCE (Figure 5B). Furthermore, ICP values tolerated for definite withdrawal were smaller compared to those for awakening trials (17.3 vs. 22.1 mmHg), and patients were required to stay below the threshold for several hours (Figure 5C).

Unfortunately, there is insufficient research to support an evidence-based development of sedation protocols in SAH. To standardize treatment by mapping the current clinical practice and to identify research gaps for further trials, a systematic survey exploring and summarizing the best practice among neurointensivists seems to be a pragmatic approach. Recognizing the limitations of this approach, we think areas with general agreement within the large body of experts comprised of multiple specialties reflect clinically effective practices and individual confounding factors, such as work environment, individual bias and demographics, become less significant. Although our web-based survey targeting German neurointensivists reached a response rate comparable to previous surveys in similar populations (33), it was lower than a previous survey covering NICUs in Germany (15). Furthermore, most participants of this survey practice in academic centers. Hence, generalizability is limited, and selection/non-response bias may be present. Although inherent to the study design, we tried to minimize response bias by creating neutral open- and closed-ended questions and debating the initial design of the questionnaire with members of IGNITE with subsequent revisions. Finally, treatment protocols might differ outside German-speaking countries due to the availability of equipment and drugs.

## 5. Conclusion

In this survey among German-speaking neurointensivists on sedation protocols in SAH, we found a convincing level of agreement on the indication and monitoring of sedation, duration of prolonged sedation, and relevant biomarkers for the withdrawal of sedation. At the same time, we also describe the variance of expert opinions to identify controversial aspects on the question of duration of sedation. In the absence of sufficient research to allow evidence-based development of sedation protocols, this survey may help to standardize treatment in healthcare systems with medical standards similar to German-speaking countries by mapping the current clinical practice and identifying research gaps.

## Data availability statement

The original contributions presented in the study are included in the article/Supplementary material, further inquiries can be directed to the corresponding author.

## Ethics statement

This study was approved by the institutional Ethics Committee of Ludwig-Maximilians University Munich, and the requirement for written consent was waived (project number 20-774 KB).

## Author contributions

MS: conceptualization, methodology, investigation, visualization, formal analysis, and writing—original draft. HL: investigation, formal analysis, and writing—review and editing. AM, TP, and SK: formal analysis and writing—review and editing. KD: conceptualization, methodology, supervision, and writing—review and editing. All authors contributed to the article and approved the submitted version.

## References

[1] Maher M Schweizer TA Macdonald RL. Treatment of spontaneous subarachnoid hemorrhage: guidelines and gaps. Stroke. (2020) 2020:1326–32. 10.1161/STROKEAHA.119.025997 31964292

[2] Etminan N Chang HS Hackenberg K De Rooij NK Vergouwen MDI Rinkel GJE. Worldwide incidence of aneurysmal subarachnoid hemorrhage according to region, time period, blood pressure, and smoking prevalence in the population: a systematic review and meta-analysis. JAMA Neurol. (2019) 76:588–97. 10.1001/jamaneurol.2019.0006 30659573 PMC6515606

[3] Venkatasubba Rao CP Suarez JI Martin RH Bauza C Georgiadis A Calvillo E. Global survey of outcomes of neurocritical care patients: analysis of the prince study part 2. Neurocrit Care. (2020) 32:88–103. 10.1007/s12028-019-00835-z 31486027

[4] Macdonald RL Schweizer TA. Spontaneous subarachnoid haemorrhage. Lancet. (2017) 389:655–66. 10.1016/S0140-6736(16)30668-7 27637674

[5] de Oliveira Manoel AL Goffi A Marotta TR Schweizer TA Abrahamson S Macdonald RL. The critical care management of poor-grade subarachnoid haemorrhage. Crit Care. (2016) 20:1–19. 10.1186/s13054-016-1193-9 26801901 PMC4724088

[6] Zoerle T Lombardo A Colombo A Longhi L Zanier ER Rampini P. Intracranial pressure after subarachnoid hemorrhage. Crit Care Med. (2015) 43:168–76. 10.1097/CCM.0000000000000670 25318385

[7] Heuer GG Smith MJ Elliott JP Winn HR Leroux PD. Relationship between intracranial pressure and other clinical variables in patients with aneurysmal subarachnoid hemorrhage. J Neurosurg. (2004) 101:408–16. 10.3171/jns.2004.101.3.0408 15352597

[8] Hawryluk GWJ Aguilera S Buki A Bulger E Citerio G Cooper DJ. A management algorithm for patients with intracranial pressure monitoring: the Seattle International Severe Traumatic Brain Injury Consensus Conference (SIBICC). Intensive Care Med. (2019) 45:1783–94. 10.1007/s00134-019-05805-9 31659383 PMC6863785

[9] Connolly ES Rabinstein AA Carhuapoma JR Derdeyn CP Dion J Higashida RT. Guidelines for the management of aneurysmal subarachnoid hemorrhage: A guideline for healthcare professionals from the american heart association/american stroke association. Stroke. (2012) 43:1711–37. 10.1161/STR.0b013e3182587839 22556195

[10] Kunze E Pham M Raslan F Stetter C Lee J-Y Solymosi L. Value of Perfusion CT, Transcranial doppler sonography, and neurological examination to detect delayed vasospasm after aneurysmal subarachnoid hemorrhage. Radiol Res Pract. (2012) 2012:1–6. 10.1155/2012/231206 23050146 PMC3462401

[11] Steiner T Juvela S Unterberg A Jung C Forsting M Rinkel G. European stroke organization guidelines for the management of intracranial aneurysms and subarachnoid haemorrhage. Cerebrovasc Dis. (2013) 35:93–112. 10.1159/000346087 23406828

[12] Diringer MN Bleck TP Hemphill JC Menon D Shutter L Vespa P. Critical care management of patients following aneurysmal subarachnoid hemorrhage: Recommendations from the neurocritical care society's multidisciplinary consensus conference. Neurocrit Care. (2011) 15:211–40. 10.1007/s12028-011-9605-9 21773873

[13] Stevens RD Naval NS Mirski MA Citerio G Andrews PJ. Intensive care of aneurysmal subarachnoid hemorrhage: an international survey. Intensive Care Med. (2009) 35:1556–66. 10.1007/s00134-009-1533-1 19533089

[14] Sakowitz OW Raabe A Vucak D Kiening KL Unterberg AW. Contemporary management of aneurysmal subarachnoid hemorrhage in Germany: results of a survey among 100 neurosurgical departments. Neurosurgery. (2006) 58:137–45. 10.1227/01.NEU.0000194532.47239.7C 16385338

[15] Hernández-Durán S Salfelder C Schaeper J Moerer O Rohde V Mielke D. von der Brelie C. Mechanical ventilation, sedation and neuromonitoring of patients with aneurysmal subarachnoid hemorrhage in Germany: results of a nationwide survey. Neurocrit Care. (2021) 34:236–47. 10.1007/s12028-020-01029-8 32583194 PMC7314429

[16] Oddo M Crippa IA Mehta S Menon D Payen JF Taccone FS Citerio G. Optimizing sedation in patients with acute brain injury. Crit Care. (2016) 2016:20. 10.1186/s13054-016-1294-5 27145814 PMC4857238

[17] Citerio G Cormio M. Sedation in neurointensive care: Advances in understanding and practice. Curr Opin Crit Care. (2003) 9:120–6. 10.1097/00075198-200304000-00007 12657974

[18] Svedung Wettervik T Howells T Lewén A Ronne-Engström E Enblad P. Temporal Dynamics of ICP, CPP, PRx, and CPPopt in High-Grade Aneurysmal Subarachnoid Hemorrhage and the Relation to Clinical Outcome. Neurocrit Care. (2021) 34:390–402. 10.1007/s12028-020-01162-4 33420669 PMC8128752

[19] Carra G Elli F Ianosi B Flechet M Huber L Rass V. Association of dose of intracranial hypertension with outcome in subarachnoid hemorrhage. Neurocrit Care. (2021) 34:722–30. 10.1007/s12028-021-01221-4 33846900

[20] Carney N Totten AM O'Reilly C Ullman JS Hawryluk GWJ Bell MJ. Guidelines for the management of severe traumatic brain injury, fourth edition. Neurosurgery. (2017) 80:6–15. 10.1227/NEU.0000000000001432 27654000

[21] Kelly DF Goodale DB Williams J Herr DL Chappell ET Rosner MJ. Propofol in the treatment of moderate and severe head injury: a randomized, prospective double-blinded pilot trial. J Neurosurg. (1999) 90:1042–52. 10.3171/jns.1999.90.6.1042 10350250

[22] Rosenow F Weber J. S2k guidelines: status epilepticus in adulthood: Guidelines of the German Society for Neurology. Nervenarzt. (2021) 92:1002–30. 10.1007/s00115-020-01036-2 33751150 PMC8484257

[23] Alldredge BK Gelb AM Isaacs SM Corry MD Allen F Ulrich S. A comparison of lorazepam, diazepam, and placebo for the treatment of out-of-hospital status epilepticus. N Engl J Med. (2001) 345:631–7. 10.1056/NEJMoa002141 11547716

[24] Said M Gümüs M Herten A Dinger TF Chihi M Darkwah Oppong M. Subarachnoid Hemorrhage Early Brain Edema Score (SEBES) as a radiographic marker of clinically relevant intracranial hypertension and unfavorable outcome after subarachnoid hemorrhage. Eur J Neurol. (2021) 28:4051–9. 10.1111/ene.15033 34293828

[25] Claassen J Carhuapoma JR Kreiter KT Du EY Connolly ES Mayer SA. Global cerebral edema after subarachnoid hemorrhage: Frequency, predictors, and impact on outcome. Stroke. (2002) 33:1225–32. 10.1161/01.STR.0000015624.29071.1F 11988595

[26] Helbok R Kurtz P Schmidt MJ Stuart MR Fernandez L Connolly SE. Effects of the neurological wake-up test on clinical examination, intracranial pressure, brain metabolism and brain tissue oxygenation in severely brain-injured patients. Crit Care. (2012) 16:R226. 10.1186/cc11880 23186037 PMC3672610

[27] Esnault P Montcriol A D'Aranda E Bordes J Goutorbe P Boret H. Early neurological wake-up test in intubated brain-injured patients: A long-term, single-centre experience. Aust Crit Care. (2017) 30:273–8. 10.1016/j.aucc.2016.10.002 27856146

[28] Skoglund K Enblad P Marklund N. Monitoring and sedation differences in the management of severe head injury and subarachnoid hemorrhage among neurocritical care centers. J Neurosci Nurs. (2013) 45:360–8. 10.1097/JNN.0b013e3182a3cf4f 24217146

[29] Robinson BRH Berube M Barr J Riker R Gélinas C. Psychometric analysis of subjective sedation scales in critically ill adults. Crit Care Med. (2013) 2013:41. 10.1097/CCM.0b013e3182a16879 23989092

[30] Deogaonkar A Gupta R DeGeorgia M Sabharwal V Gopakumaran B Schubert A. Bispectral Index monitoring correlates with sedation scales in brain-injured patients. Crit Care Med. (2004) 32:2403–6. 10.1097/01.CCM.0000147442.14921.A5 15599143

[31] Jung JY Cho CB Min BM. Bispectral index monitoring correlates with the level of consciousness in brain injured patients. Korean J Anesthesiol. (2013) 64:246–50. 10.4097/kjae.2013.64.3.246 23560191 PMC3611075

[32] Olson DM Thoyre SM Peterson ED Graffagnino C A. randomized evaluation of bispectral index-augmented sedation assessment in neurological patients. Neurocrit Care. (2009) 11:20–7. 10.1007/s12028-008-9184-6 19184556 PMC2706915

[33] Murphy SA Bell MJ Clark ME Whalen MJ Noviski N. Pediatric Neurocritical Care: A Short Survey of Current Perceptions and Practices. Neurocrit Care. (2015) 23:149–58. 10.1007/s12028-015-0120-2 25693892

