# Supplementary material for: Sedation protocols in non-traumatic SAH (SPRINT-SAH): A cross-sectional survey among German-speaking neurointensivists
Source: Front Neurol. 2023 Feb 13;14:1058804. doi: 10.3389/fneur.2023.1058804 (PMC9969111; doi:10.3389/fneur.2023.1058804)
Supplement: Supplementary file 1 [file Data_Sheet_1.docx]

Supplementary Material

# Sedatives and multi-modal neuromonitoring used during prolonged sedation

(A, n = 37, multiple selection) Sedatives and analgetics used for initial (<48 hours) and prolonged sedation (> 48 hours). (B, n = 37, multiple selection) Multi-modal neuromonitoring methods used during sedation of SAH patients.

*PICCO* Pulse Contour Cardiac Output, *etCO_2_* endtidal carbon dioxide, *CVP* central venous pressure, *PtbO_2_* brain tissue oxygen tension, *SjvO_2_* jugular venous oxygen saturation, *cEEG* continuous electroencephalography, *CT* computer tomography, *EVD* external ventricular drain

# Questionnaire

**German Version**

1. Bitte geben Sie Ihr Geburtsjahr an.
2. Über welche Versorgungstufe verfügt das Krankenhaus, in dem Sie aktuell tätig sind?
   1. Universitätsklinik
   2. Klinik der Maximal/Schwerpunktversorgung
   3. Klinik der Grund- und Regelversorgung
   4. Unfallklinik
   5. Rehaklinik
3. Über wie viele stationäre Betten verfügt Ihre Einrichtung?
4. Wie viele Fälle nicht-traumatischer Subarachnoidalblutungen (SABs, ICD 10 I60.X) behandelt Ihre Institution im Jahr?
5. Über wie viele Jahre Berufserfahrung verfügen Sie (seit Beginn ärztlicher Tätigkeit)?
6. Bitte geben sie Ihre Erfahrung in der Intensivmedizin an (Jahre).
7. Welcher Fachrichtung gehören Sie an?
   1. Neurologie
   2. Neurochirurgie
   3. Anästhesie
   4. Innere Medizin
   5. Sonstige
8. Über welches Organisationsmodell verfügt Ihre auf Hirnschädigungen spezialisierte Intensivstation?
   1. Rein neurologisch geführte Intensivstation
   2. Rein neurochirurgisch geführte Intensivstation
   3. Interdisziplinär neurologisch/neurochirurgisch geführte Intensivstation
   4. Interdisziplinär anästhesiologisch/neurologisch geführte Intensivstation
   5. Interdisziplinär anästhesiologisch/neurochirurgisch geführte Intensivstation
   6. Interdisziplinär internistisch/neurologisch geführte Intensivstation
   7. Interdisziplinäre Intensivstation mit neurologisch/neurochirurgischen Betten
   8. Sonstige
9. Bitte beschreiben Sie das Organisationsmodell Ihrer auf Hirnschädigung spezialisierten Intensivstation.
10. Ist eine standard operating procedure (SOP)/Richtlinie für die Behandlung von SAB Patienten in Ihrer Institution etabliert?
    1. Ja
    2. Nein
11. Ist eine standard operating procedure (SOP)/Richtlinie für die Sedierung von SAB Patienten in Ihrer Institution etabliert?
    1. Ja
    2. Nein
12. Welche Indikationen zur prolongierten Sedierung bei Neurointensivpatienten werden in Ihrer Institution grundsätzlich angewendet? (Mehrfachnennung möglich)
    1. ICP Kontrolle
    2. Temperatur Zielsetzung
    3. Behandlung von Status epilepticus
    4. Angiographischer Vasospasmus
    5. Sympaticus Hyperaktivität / Agitation
    6. Ventilator Asynchronität
    7. Sonstige
13. Bitte definieren Sie sonstige Indikationen für eine prolongierte Sedierung.
14. Verwenden Sie klinische Skalen wie WFNS, Hunt und Hess, Fisher usw., um bei Aufnahme von SAB-Patienten die Indikationsstellung sowie die Dauer einer prolongierten Sedierung zu evaluieren?
    1. Ja
    2. Nein
15. Welchen Biomarker erachten Sie neben den etablierten klinischen Skalen (WFNS, Hunt und Hess etc.) bei SAB-Patienten für die Indikationsstellung sowie die Dauer einer prolongierten Sedierung am wichtigsten?
    1. Initiale therapierefraktäre ICP Krise
    2. Radiologischer Nachweis eines erhöhten intrakraniellen Drucks durch Parenchym- oder Blutkompartiment (zB globales cerebrales Ödem, Parenchymhämatom)
    3. Re-Blutung innerhalb der ersten 72 Stunden
    4. Akuter Hydrocephalus
    5. Akut symptomatischer Anfall bei Blutungsereignis
    6. Kritische Vasospasmen (MCA/ACI Index >3)
    7. Sonstige
16. Welchen Biomarker erachten Sie neben den etablierten klinischen Skalen (WFNS, Hunt und Hess etc.) bei SAB-Patienten für die Indikationsstellung sowie die Dauer einer prolongierten Sedierung am zweitwichtigsten?
    1. Initiale therapierefraktäre ICP Krise
    2. Radiologischer Nachweis eines erhöhten intrakraniellen Drucks durch Parenchym- oder Blutkompartiment (zB globales cerebrales Ödem, Parenchymhämatom)
    3. Re-Blutung innerhalb der ersten 72 Stunden
    4. Akuter Hydrocephalus
    5. Akut symptomatischer Anfall bei Blutungsereignis
    6. Kritische Vasospasmen (MCA/ICA Index >3)
    7. Sonstige
17. Welchen Biomarker erachten Sie neben den etablierten klinischen Skalen (WFNS, Hunt und Hess etc.) bei SAB-Patienten für die Indikationsstellung sowie die Dauer einer prolongierten Sedierung am drittwichtigsten?
    1. Initiale therapierefraktäre ICP Krise
    2. Radiologischer Nachweis eines erhöhten intrakraniellen Drucks durch Parenchym- oder Blutkompartiment (zB globales cerebrales Ödem, Parenchymhämatom)
    3. Re-Blutung innerhalb der ersten 72 Stunden
    4. Akuter Hydrocephalus
    5. Akut symptomatischer Anfall bei Blutungsereignis
    6. Kritische Vasospasmen (MCA/ICA Index >3)
    7. Sonstige
18. Führen Sie standardisierte und regelmäßige Aufwachversuche (awakening trials) bei SAB Patienten durch, die nach Ihren Kriterien die Indikation für eine prolongierte Sedierung erfüllen?
    1. Ja
    2. Nein
19. Wie wird die Entscheidung hinsichtlich des Zeitpunkts des endgültigen Sedierungsauslasses gefällt?
    1. Interdisziplinärer Konsensus ärztlich, Einzelfall
    2. Multi-professionell, Einzelfall
    3. Oberarzt der Station, Einzelfall
    4. Standardisiert nach Kriterien der internen SOP
20. Existieren prä-definierte Kriterien für den Abbruch des Sedierungsauslasses?
    1. Ja
    2. Nein
21. Was ist der maximal tolerierte intrakranielle Druck während eines Sedierungsauslasses?
22. Was ist die minimal tolerierte Sauerstoffsättigung während eines Sedierungsauslasses?
23. Welche der folgenden Komplikationen würde Ihrer Meinung nach den Abbruch eines Sedierungsauslasses gerechtfertigten (Mehrfachnennung möglich)?
    1. Verschlechterung des Bewusstseins, neue fokal-neurologische Defizite
    2. Nicht kontrollierbare vegetative Stressreaktion (Hypertonie, Tachykardie, psychomotorische Unruhe)
    3. Neuer duplexsonographischer Vasospasmus ohne korrespondierendes neurologisches Defizit (MCA/ICA Index >3)
    4. Zunahme der bildmorphologischen Korrelate eines Hirndrucks (z.B. globales cerebrales Ödems)
    5. Sonstige
24. Für welche Zeit würden Sie eine vegetative Stressreaktion während eines Sedierungsauslasses tolerieren?
25. Bitte spezifizieren sie sonstige Komplikationen, die Sie zu einem Abbruch des Sedierungsauslasses bewegen würden.
26. Welche Monitoringverfahren werden zur Steuerung der Sedierungstiefe verwendet, wenn eine prolongierte Sedierung bei SAB Patienten erfolgt (Mehrfachnennung möglich)?
    1. Klinische Untersuchung, Richmond Agitation Sedation Scale (RASS)
    2. EEG
    3. Sonstige
27. Bitte definieren Sie sonstige Monitoringverfahren zur Bestimmung der Sedierungstiefe.
28. Welche therapeutischen Zielwerte streben Sie an, wenn eine prolongierte Sedierung bei SAB Patienten erfolgt?
    1. RASS -5, Husten/Spontanatmung wird toleriert
    2. RASS -5, kein Hustenreflex, keine Spontanatmung

c. RASS -4

- d. RASS -3/-2
- e. RASS -1

29. Welches EEG-Verfahren wird in Ihrer Institution hauptsächlich verwendet (Mehrfachnennung möglich)?

1. Mehrkanal EEG
2. Bispektraler Index (BIS)

30. Wird ein therapeutischer Zielwert zur Steuerung der Sedierungstiefe durch EEG/BIS festgelegt?

1. Ja
2. Nein

31. Bitte geben Sie den therapeutischen Zielwert an (BIS).

32. Bitte geben Sie den therapeutischen Zielwert der burst suppression ratio an.

33. Welche der folgenden Sedativa nutzen Sie primär für die initiale Sedierung (< 48h) von SAB Patienten (Mehrfachnennung möglich)?

a.Propofol

b. (Es)ketamin

c. Midazolam

d. Thiopental

e. Methohexital

f. Isoflurane

g. Desflurane

h. Clonidin

i. Dexmedetomidine

j. Opiate

34. Welche der folgenden Sedativa nutzen Sie primär für die prolongierte Sedierung (>48h) von SAB Patienten (Mehrfachnennung möglich)?

a. Propofol

b. (Es)ketamin

c. Midazolam

d. Thiopental

e. Methohexital

f. Isoflurane

g. Desflurane

h. Clonidin

i. Dexmedetomidine

j. Opiate

35. Welche der folgenden Monitoringverfahren nutzen Sie regelmäßig bei SAB Patienten (Mehrfachnennung möglich)?

a. Thermodilution/Pulskonturanalyse (z.B. PICCO)

b. Zentraler Venendruck (CVP)

c. Endtidales CO2 (et CO2)

d. zerebraler Sauerstoffpartialdruck (Pbt O2)

e. jugularvenöse Sauerstoffmessung (SjvO2)

f. Routinemäßige CT-Angiographie / CT-Perfusion (CTA/CTP)

g. Messung der cerebralen Autoregulation

h. Microdialyse

i. Quantitatives cEEG

j. Duplex-Sonographie

k. ICP-Parenchymsonde

l. EVD offen (>15min/60min)

m. EVD geschlossen (>45min/60min)

n. Sonstge

36. Bitte definieren Sie sonstige Monitoringverfahren.

37. Bestimmen Sie bei Aufnahme von SAB Patienten anhand der vorliegenden Biomarker, ein zeitliches Intervall, über das hinaus eine prolongierte Sedierung mindestens aufrechterhalten werden soll?

a. Ja

b. Nein

38. Länge der prolongierten Sedierung anhalt der Anwesenheit/Abwesenheit des wichtigsten Biomarkers sowie anhand von 1,2, oder 3 ungünstigen Biomarkers bei good grade SAB Patienten (WFNS 1-3).

39. Länge der prolongierten Sedierung anhalt der Anwesenheit/Abwesenheit des wichtigsten Biomarkers sowie anhand von 1,2, oder 3 ungünstigen Biomarkers bei poor grade SAB Patienten (WFNS 4-5).

40. Würden Sie anders entscheiden, wenn eine chirurgische anstatt einer endovaskulären Aneurysmaversorgung erfolgt wäre?

1. Ja
2. Nein

41. Würden Sie anders entscheiden, wenn initial mittels CT A/DSA kein Aneurysmanachweis gelungen wäre (nicht-traumatische, nicht-aneurysmatische SAB ohne peri-mesencephale Blutverteilung)?

1. Ja
2. Nein

42. Sehen Sie anhaltende Vasospasmen (duplexsonographisch, MCA/ICA Index >3) als Kontraindikation für einen Sedierungsauslass?

1. Ja
2. Nein

43. Welchen Schwellenwert legen Sie für den MCA/ICA Index fest, um einen Sedierungsauslass durchzuführen?

44. Welchen Schwellenwert streben Sie für den ICP an, um nach initialer therapierefraktärer ICP Krise einen Sedierungsauslass durchführen zu können?

45. Für wie lange sollte der tatsächliche ICP unter oben genanntem Schwellenwert liegen, um nach initialer therapierefraktärer ICP Krise einen Sedierungsauslass durchführen zu können?

46. Erfolgt bei Nachweis von erhöhtem intrakraniellem Druck durch radiologische Korrelate (z.B. globales cerebrales Ödem, Herniation, Parenchymhämatom) eine erneute bildgebende Kontrolle vor einem Sedierungsauslass?

1. Ja
2. Nein

47. Welche bildgebenden Parameter müssen mindestens erfüllt sein, um einen Sedierungsauslass durchzuführen?

1. Globales cerebrales Ödem rückläufig, radiologische Korrelate eines erhöhten Hirndruckes weiterhin vorhanden (durch Ödem, territoriale Ischämien oder ICB)
2. KEIN globales cerebrales Ödem, KEINE raumfordernden territorialen Ischämien, KEINE raumfordernde ICB
3. keine bildgebenden Korrelate einer Herniation oder erhöhten intrakraniellen Drucks durch Blut- oder Parenchymkomponente

**English Translation**

1. Please provide your year of birth.
2. Which level of care does your current hospital provide?
   1. University hospital
   2. Maximum capacity hospital
   3. Medium capacity hospital
   4. Trauma hospital
   5. Rehabilitation hospital
3. How many hospital beds does your hospital provide?
4. How many cases of non-traumatic subarachnoid hemorrhage (SABs, ICD 10 I60.X) does your institution treat annually?
5. How many years have you been a licensed physician (since start of clinical practice)?
6. Please provide the number of years of experience in intensive care medicine.
7. Please provide your subspecialty.
   1. Neurology
   2. Neurosurgery
   3. Anesthesiology
   4. Internal medicine
   5. Other
8. How is the ICU at your current institution for the treatment of SAH patients structured?
   1. Neurology led ICU
   2. Neurosurgery led ICU
   3. Interdisciplinary neurology/neurosurgery led ICU
   4. Interdisciplinary anesthesiology/neurology led ICU
   5. Interdisciplinary anesthesiology/neurosurgery led ICU
   6. Interdisciplinary internal medicine/neurology led ICU
   7. Interdisciplinary with neurology and neurosurgery
   8. Other
9. Please describe the organizational structure of your specialized ICU.
10. Does your institution have an established standard operating procedure (SOP)/guideline for the general management of SAH patients?
    1. Yes
    2. No
11. Does your institution have an established standard operating procedure (SOP)/guideline for the sedation of SAH patients?
    1. Yes
    2. No
12. Which indication for prolonged sedation of neurointensive care patients does your institution generally apply (multiple answers possible)?
    1. Intracranial pressure (ICP) control
    2. Targeted temperature management
    3. Therapy of status epilepticus
    4. Angiographically proven vasospasm
    5. Sympathetic hyperactivity/agitation
    6. Ventilator asynchrony
    7. Other
13. Please define further indications for prolonged sedation at your institution.
14. Are clinical scales (WFNS, Hunt and Hess, Fisher etc.) used upon admission to evaluate the indication/duration of prolonged sedation at your institution?
    1. Yes
    2. No
15. Which biomarkers, aside from clinical scales (WFNS, Hunt and Hess, Fisher etc.) would you rank most important for the indication/duration of prolonged sedation in SAH patients?
    1. Therapy refractory ICP elevation
    2. Radiographic features of elevated ICP (cerebral edema, parenchymal hematoma)
    3. Recurrent bleeding within 72 hours
    4. Acute hydrocephalus
    5. Acute symptomatic seizure
    6. Critical vasospasm (MCA/ICA Index >3)
    7. Other
16. Which biomarkers, aside from clinical scales (WFNS, Hunt and Hess, Fisher etc.) would you rank second most important for the indication/duration of prolonged sedation in SAH patients?
    1. Therapy refractory ICP elevation
    2. Radiographic features of elevated ICP (cerebral edema, parenchymal hematoma)
    3. Recurrent bleeding within 72 hours
    4. Acute hydrocephalus
    5. Acute symptomatic seizure
    6. Critical vasospasm (MCA/ICA Index >3)
    7. Other
17. Which biomarkers, aside from clinical scales (WFNS, Hunt and Hess, Fisher etc.) would you rank third most important for the indication/duration of prolonged sedation in SAH patients?
    1. Therapy refractory ICP elevation
    2. Radiographic features of elevated ICP (cerebral edema, parenchymal hematoma)
    3. Recurrent bleeding within 72 hours
    4. Acute hydrocephalus
    5. Acute symptomatic seizure
    6. Critical vasospasm (MCA/ICA Index >3)
    7. Other
18. Are awakening trials regularly performed in SAH patients meeting indication for prolonged sedation at your institution?
    1. Yes
    2. No
19. How ist the decision regarding the time point to stop prolonged sedation indefinitely made at your institution?
    1. Interdisciplinary consensus among physicians and individually case based
    2. Multiprofessional and individually case based
    3. Attending physician and individually case based
    4. Standardized according to institutional SOP
20. Do pre-existing criteria to end an awakening trial exist at your institution?
    1. Yes
    2. No
21. What is the maximum threshold of intracranial pressure (ICP) tolerated during an awakening trial?
22. What is the minimum oxygen saturation tolerated during an awakening trial?
23. In your opinion, which of the following complications would warrant discontinuation of an awakening trial (multiple answers possible)?
    1. New focal signs or reduced consciousness
    2. Vegetative stress reaction (hypertension, tachycardia, restlessness)
    3. New vasospasm on doppler sonography without corresponding focal deficits (MCA/ICA Index >3)
    4. Increase in radiographic features indicating elevated ICP
    5. Other
24. What ist the maximum duration of a vegetative stress reaction you would tolerate during an awakening trial?
25. Please specify further complications warranting discontinuation of an awakening trial.
26. Which methods are used for therapeutic monitoring of sedation depth in patients with SAH at your institution?
    1. Clinical exam, Richmond Agitation Sedation Scale (RASS)
    2. EEG
    3. Other
27. Please specify further methods used for therapeutic monitoring of sedation depth in patients with SAH at your institution.
28. Which target RASS value is applied for prolonged sedation of SAH patients at your institution?
    1. RASS -5, cough reflex/spontaneous breaths tolerated
    2. RASS -5, no cough reflex or spontaneous breaths tolerated

c. RASS -4

- d. RASS -3/-2
- e. RASS -1

1. Which EEG technique is mainly used at your institution (multiple answers possible)?
2. Multiple channel EEG
3. Bispectral Index (BIS)
4. Are therapeutic target values derived from EEG/BIS applied to guide the depth of sedation at your institution?

a. Yes

b. No

1. Please provide the therapeutic target BIS value.
2. Please provide the therapeutic target value of the burst suppression ratio.
3. Which of the following sedatives are primarily used for the initial sedation (<48h) of SAH patients at your institution (multiple answers possible)?

a. Propofol

b. (Es)ketamin

c. Midazolam

d. Thiopental

e. Methohexital

f. Isoflurane

g. Desflurane

h. Clonidin

i. Dexmedetomidine

j. Opioids

1. Which of the following sedatives are primarily used for prolonged sedation (>48h) of SAH patients at your institution (multiple answers possible)?

a. Propofol

b. (Es)ketamin

c. Midazolam

d. Thiopental

e. Methohexital

f. Isoflurane

g. Desflurane

h. Clonidin

i. Dexmedetomidine

j. Opioids

1. Which of the following methods are used regularly to monitor SAH patients at your institution (multiple answers possible)?

a. Thermodilution/Pulse contour analysis (PICCO)

b. Central venous pressure (CVP)

c. Endtidal CO2 (et. CO2)

d. Cerebral oxygen saturation (Pbt 02)

e. Jugular venous oxygen saturation (sjv02)

f. Routine CT-Angiography/ CT Perfusion (CTA/CTP)

g. Measurement of cerebral oxygen autoregulation

h. Microdialysis

i. Quantitative cEEG

j. Duplex sonography

k. Intraparenchymal pressure monitor

l. EVD (open >15minutes/60 minutes)

m. EVD (closed >45minutes/60 minutes)

n. Other

1. Please define further monitoring methods used at your institution.
2. Do you decide on a minimum duration of prolonged sedation for SAH patients upon admission based on predefined biomarkers at your institution?

a. Yes

b. No

1. Duration of prolonged sedation in days based on the presence/absence of the most important biomarker as well as presence of 1,2, or 3 unfavorable biomarkers in good grade SAH (WFNS 1-3) patients.
2. Duration of prolonged sedation in days based on the presence/absence of the most important biomarker as well as presence of 1,2, or 3 unfavorable biomarkers in poor grade SAH (WFNS 4-5) patients.
3. Would your decision have changed if your SAH patient had received surgical rather than endovascular management of a cerebral aneurysm?
4. Yes
5. No
6. Would your decision have changed if an aneurysm was not found on initial CT-A/DSA (non-traumatic, non-aneurysmal SAH without peri-mesencephalic bleeding)
7. Yes
8. No
9. Do you view continuous vasospasm (duplex sonographic, MCA/ICA Index >3) as a contraindication for an awakening trial?
10. Yes
11. No
12. At which threshold value of the MCA/ICA Index would you perform an awakening trial?
13. Which threshold value of ICP would you target in order to safely perform an awakening trial following a therapy refractory crisis of increased ICP?
14. For how long would the actual ICP value need to be below the above mentioned threshold value, in order to safely perform an awakening trial following a therapy refractory crisis of increased ICP?
15. Would you perform repeat radiographic imaging prior to an awakening trial if signs of increased intracranial pressure (global cerebral edema, herniation, parenchymal hematoma) had been demonstrated previously?
16. Yes
17. No
18. What are the minimum radiographic features to be fulfilled in order to perform an awakening trial?

a. Regression of global cerebral edema, radiographic features of increased ICP remaining (edema, territorial ischemia or intracranial hemorrhage)

b. NO global cerebral edema, NO space occupying territorial ischemia, NO space occupying ICH

c. NO radiographic features of herniation or increased ICP caused by hemorrhagic or parenchymal components

# Scores

# Richmond Agitation Sedation Scale (RASS)

+4 Combative

+3 Very agitated

+2 Agitated

+1 Restless

0 Alert and calm

-1 Drowsy

-2 Light sedation

-3 Moderate sedation

-4 Deep sedation

-5 Unarousable

# Hunt and Hess grading system

Grade I Asymptomatic, or minimal headache and slight nuchal rigidity.

Grade II Moderate to severe headache, nuchal rigidity, no neurological deficit other than cranial nerve palsy.

Grade III Drowsiness, confusion, or mild focal deficit.

Grade IV Stupor, moderate to severe hemiparesis, possibly early decerebrate rigidity and vegetative disturbances.

Grade V Deep coma, decerebrate rigidity, moribund appearance.

# WFNS (World Federation of Neurological Surgeons) grading scale

Grade I: GCS 15

Grade II: GCS 13-14, without focal neurological deficit

Grade III: GCS 13-14, with focal neurological deficit

Grade IV: GCS 7-12

Grade V: GCS 3-6
